# *rac*-3-(4-Chloro­phen­yl)-3a,4-di­hydro-3*H*-chromeno[4,3-*c*]isoxazole-3a-carbo­nitrile

**DOI:** 10.1107/S1600536813009653

**Published:** 2013-04-13

**Authors:** S. Paramasivam, J. Srinivasan, P.R. Seshadri, M. Bakthadoss

**Affiliations:** a Post Graduate and Research Department of Physics, Agurchand Manmull Jain College, Chennai 600 114, India; b Department of Organic Chemistry, University of Madras, Guindy Campus, Chennai 600 025, India

## Abstract

The title compound, C_17_H_11_ClN_2_O_2_, which contains two stereogenic C atoms, crystallizes in a centrosymmetric space group as a racemate. The pyran ring and the isoxazole ring adopt sofa and twisted conformations, respectively. The dihedral angle between the benzene ring and the mean plane through the near coplanar atoms of the pyran ring is 4.17 (5)°. The mol­ecular conformation features a weak C—H⋯O contact. In the crystal, C—H⋯O hydrogen bonds link the mol­ecules, forming chains along the *a*-axis direction.

## Related literature

For the biological activity of isoxazole derivatives, see: Mullen *et al.* (1988 ▶); Eddington *et al.* (2002 ▶); Kashiwada *et al.* (2001 ▶); Caine (1993 ▶). For a related structure, see: Paramasivam *et al.* (2012 ▶). For conformational analysis and pukering parameters, see: Cremer & Pople (1975 ▶).
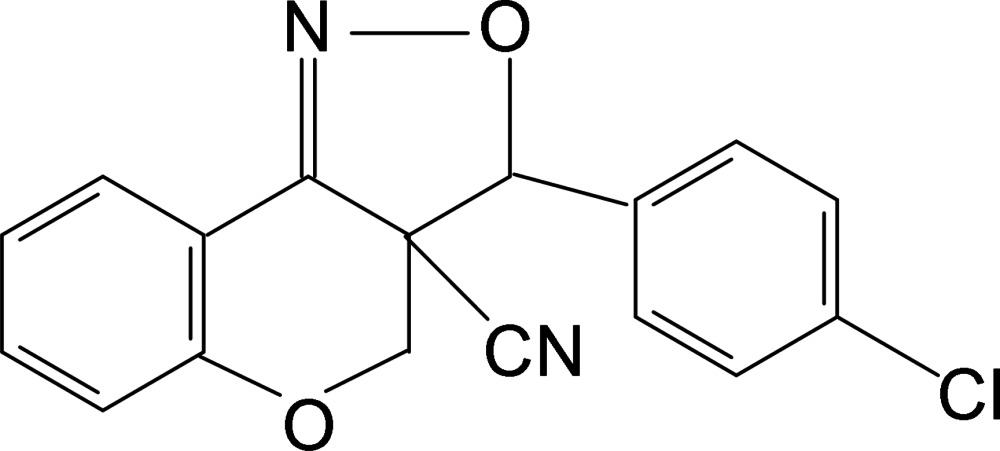


## Experimental

### 

#### Crystal data

C_17_H_11_ClN_2_O_2_
*M* *_r_* = 310.73
Monoclinic, 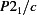

*a* = 6.7891 (2) Å
*b* = 13.9921 (3) Å
*c* = 15.1788 (3) Å
β = 101.175 (1)°
*V* = 1414.55 (6) Å^3^
*Z* = 4
Mo *K*α radiation
μ = 0.28 mm^−1^
*T* = 298 K
0.30 × 0.25 × 0.20 mm

#### Data collection

Bruker SMART APEXII area-detector diffractometer
Absorption correction: multi-scan (*SADABS*; Bruker, 2008 ▶) *T*_min_ = 0.921, *T*_max_ = 0.946
13620 measured reflections
3541 independent reflections
2865 reflections with *I* > 2σ(*I*)
*R*_int_ = 0.021

#### Refinement

*R*[*F* ^2^ > 2σ(*F* ^2^)] = 0.042
*wR*(*F* ^2^) = 0.132
*S* = 1.00
3541 reflections
199 parameters
H-atom parameters constrained
Δρ_max_ = 0.24 e Å^−3^
Δρ_min_ = −0.37 e Å^−3^

### 

Data collection: *APEX2* (Bruker, 2008 ▶); cell refinement: *SAINT* (Bruker, 2008 ▶); data reduction: *SAINT*; program(s) used to solve structure: *SHELXS97* (Sheldrick, 2008 ▶); program(s) used to refine structure: *SHELXL97* (Sheldrick, 2008 ▶); molecular graphics: *ORTEP-3 for Windows* (Farrugia, 2012 ▶) and *PLATON* (Spek, 2009 ▶); software used to prepare material for publication: *SHELXL97*, *PLATON* and *publCIF* (Westrip, 2010 ▶).

## Supplementary Material

Crystal structure: contains datablock(s) I, global. DOI: 10.1107/S1600536813009653/kp2448sup1.cif

Structure factors: contains datablock(s) I. DOI: 10.1107/S1600536813009653/kp2448Isup2.hkl

Supplementary material file. DOI: 10.1107/S1600536813009653/kp2448Isup3.cml

Additional supplementary materials:  crystallographic information; 3D view; checkCIF report

## Figures and Tables

**Table 1:** Hydrogen-bond geometry (Å, °)

*D*—H⋯*A*	*D*—H	H⋯*A*	*D*⋯*A*	*D*—H⋯*A*
C13—H13⋯O1	0.93	2.42	2.7733 (19)	102
C5—H5⋯O2^i^	0.93	2.47	3.3422 (19)	156

Symmetry code: (i) 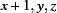.

**Fig. 1.**
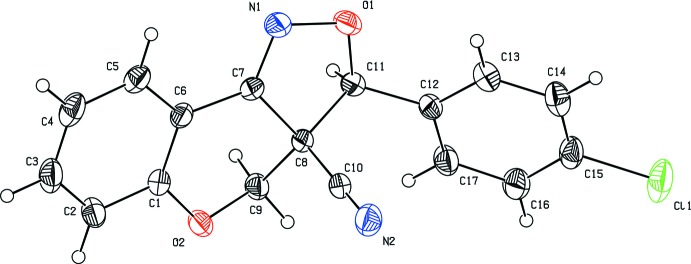
The molecular structure of the title compound, showing the atom-numbering scheme and displacement ellipsoids drawn at the 20% probability level.

## supplementary crystallographic information

### Comment

As a continuation of our research related to isoxazole containing chromenoisoxazole moiety, we analysed the crystal structure of *rac*-6-Ethoxy-3,3a,4,9 b-tetrahydro-1,3- diphenyl-1*H*-chromeno[4,3-*c*]isoxazole- 3a-carbonitrile (Paramasivam *et al.*, 2012). The present compound exhibits the pronounced similarity to the previous ones, either in bond lengths and angles as well as in molecular conformations.

Isoxazole derivative exhibit anti-fungal (Mullen *et al.*, 1988) and anti-consulvant (Eddington *et al.*, 2002) activities whereas benzopyran and chromenopyrrole derivatives exhibit anti-HIV activities (Kashiwada *et al.*, 2001) and used in the treatment of impulsive-disorder disease (Caine, 1993). On these grounds, the title compound was chosen for X-ray structure analysis (Fig.1).

The pyran ring (O2/C1/C6—C9) adopts a sofa conformation with the puckering parameters (Cremer & Pople, 1975) being q_2_=0.359 (1) Å, q_3_=-0.292 (1) Å, Q_T_=0.463 (1) Å and the five-membered isoxazole ring (N1/O1/C7/C8/C11) adopts an envelope conformation with puckering parameters (Cremer & Pople, 1975) being q_2_=0.284 (1) Å and Φ_2_=142.4 (3)°.

The dihedral angle between the pyran and the benzene rings (C1—C6) is 4.17 (5)°. The dihedral angle between the chromeno ring (fusion of benzene and pyran rings) and isoxazole ring is 13.42 (5)°. In the chromenoisoxazole moiety, the dihedral angle between the benzene and isoxazole ring is 10.83 (5)° and the dihedral angle between the pyran and isoxazole ring is 14.81 (5)°.

The geometric parameters of the title compound (Fig. 1) agree well with the reported ones of similar structures (Paramasivam *et al.*, 2012).

The molecular structure is stabilized by C—H···O intramolecular interaction and the crystal packing is stabilized by C—H···O hydrogen bonds (Table 1).

### Experimental

A solution of (*E*)-3-(4-chlorophenyl)-2-((2-((*E*)-(hydroxyimino)methyl)phenoxy)methyl) acrylonitrile (2 mmol) in CCl_4_ at (273–283 K) was added pinch wise NCS (4 mmol) over 3 h. After Et_3_N (4 mmol) was added the reaction mixture was stirred at room temperature for 2 h. After completion of the reaction, reaction mixture was evaporated under reduced pressure and the resulting crude mass was diluted with water (15 mL) and extracted with ethyl acetate (3 × 15 mL). The combining organic layer was washed with brine (2 × 10 mL) and dried over anhydrous Na_2_SO_4_. The organic layer was evaporated and purified by column chromatography (silica gel 60–120 mesh 7% EtOAc in hexanes) to provide the desired pure product 3-(4-chlorophenyl)-3a,4-dihydro-3*H*-chromeno[4,3-*c*]isoxazole-3a-carbonitrile a as colourless solid.

### Refinement

Hydrogen atoms were positioned geometrically and allowed to ride on their parent atoms, with C—H = 0.93 - 0.97 Å and *U*_iso_(H) = 1.5*U*_eq_(C) for methyl H atoms and 1.2 *U*_eq_(C) for other H atoms.

### Crystal data

**Table:**

C_17_H_11_ClN_2_O_2_	*F*(000) = 640
*M**_r_* = 310.73	Monoclinic
Monoclinic, *P*2_1_/*c*	*D*_x_ = 1.459 Mg m^−^^3^
Hall symbol: -P 2ybc	Mo *K*α radiation, λ = 0.71073 Å
*a* = 6.7891 (2) Å	Cell parameters from 3541 reflections
*b* = 13.9921 (3) Å	θ = 2.0–28.4°
*c* = 15.1788 (3) Å	µ = 0.28 mm^−^^1^
β = 101.175 (1)°	*T* = 298 K
*V* = 1414.55 (6) Å^3^	Block, colourless
*Z* = 4	0.30 × 0.25 × 0.20 mm

### Data collection

**Table:**

Bruker SMART APEXII area-detector diffractometer	3541 independent reflections
Radiation source: fine-focus sealed tube	2865 reflections with *I* > 2σ(*I*)
Graphite monochromator	*R*_int_ = 0.021
ω and φ scans	θ_max_ = 28.4°, θ_min_ = 2.0°
Absorption correction: multi-scan (*SADABS*; Bruker, 2008)	*h* = −9→8
*T*_min_ = 0.921, *T*_max_ = 0.946	*k* = −18→11
13620 measured reflections	*l* = −20→20

### Refinement

**Table:**

Refinement on *F*^2^	Primary atom site location: structure-invariant direct methods
Least-squares matrix: full	Secondary atom site location: difference Fourier map
*R*[*F*^2^ > 2σ(*F*^2^)] = 0.042	Hydrogen site location: inferred from neighbouring sites
*wR*(*F*^2^) = 0.132	H-atom parameters constrained
*S* = 1.00	*w* = 1/[σ^2^(*F*_o_^2^) + (0.0796*P*)^2^ + 0.2931*P*] where *P* = (*F*_o_^2^ + 2*F*_c_^2^)/3
3541 reflections	(Δ/σ)_max_ < 0.001
199 parameters	Δρ_max_ = 0.24 e Å^−^^3^
0 restraints	Δρ_min_ = −0.37 e Å^−^^3^

### Special details

**Table:**

Geometry. All e.s.d.'s (except the e.s.d. in the dihedral angle between two l.s. planes) are estimated using the full covariance matrix. The cell e.s.d.'s are taken into account individually in the estimation of e.s.d.'s in distances, angles and torsion angles; correlations between e.s.d.'s in cell parameters are only used when they are defined by crystal symmetry. An approximate (isotropic) treatment of cell e.s.d.'s is used for estimating e.s.d.'s involving l.s. planes.
Refinement. Refinement of *F*^2^ against ALL reflections. The weighted *R*-factor *wR* and goodness of fit *S* are based on *F*^2^, conventional *R*-factors *R* are based on *F*, with *F* set to zero for negative *F*^2^. The threshold expression of *F*^2^ > σ(*F*^2^) is used only for calculating *R*-factors(gt) *etc*. and is not relevant to the choice of reflections for refinement. *R*-factors based on *F*^2^ are statistically about twice as large as those based on *F*, and *R*- factors based on ALL data will be even larger.

### Fractional atomic coordinates and isotropic or equivalent isotropic displacement parameters (Å^2^)

**Table:**

	*x*	*y*	*z*	*U*_iso_*/*U*_eq_	
C1	0.7698 (2)	0.37043 (10)	0.51654 (9)	0.0382 (3)	
C2	0.7964 (3)	0.34701 (12)	0.60681 (10)	0.0497 (4)	
H2	0.6880	0.3473	0.6360	0.060*	
C3	0.9865 (3)	0.32316 (13)	0.65285 (10)	0.0565 (4)	
H3	1.0052	0.3066	0.7132	0.068*	
C4	1.1478 (3)	0.32362 (12)	0.61050 (11)	0.0551 (4)	
H4	1.2747	0.3077	0.6424	0.066*	
C5	1.1226 (2)	0.34766 (11)	0.52083 (11)	0.0457 (3)	
H5	1.2326	0.3483	0.4926	0.055*	
C6	0.9322 (2)	0.37097 (9)	0.47255 (9)	0.0360 (3)	
C7	0.89137 (18)	0.39271 (10)	0.37728 (9)	0.0350 (3)	
C8	0.67441 (18)	0.39375 (10)	0.32830 (8)	0.0330 (3)	
C9	0.5486 (2)	0.43971 (11)	0.38997 (9)	0.0402 (3)	
H9A	0.5883	0.5059	0.4008	0.048*	
H9B	0.4079	0.4384	0.3613	0.048*	
C10	0.6106 (2)	0.29492 (11)	0.30501 (8)	0.0383 (3)	
C11	0.7019 (2)	0.45257 (10)	0.24579 (9)	0.0382 (3)	
H11	0.6890	0.5206	0.2592	0.046*	
C12	0.5619 (2)	0.42986 (10)	0.15911 (8)	0.0369 (3)	
C13	0.6257 (3)	0.38630 (11)	0.08816 (10)	0.0459 (3)	
H13	0.7603	0.3701	0.0933	0.055*	
C14	0.4913 (3)	0.36634 (11)	0.00910 (11)	0.0533 (4)	
H14	0.5353	0.3378	−0.0390	0.064*	
C15	0.2925 (3)	0.38934 (12)	0.00299 (10)	0.0509 (4)	
C16	0.2244 (3)	0.43228 (14)	0.07288 (11)	0.0553 (4)	
H16	0.0891	0.4470	0.0679	0.066*	
C17	0.3603 (2)	0.45313 (13)	0.15060 (10)	0.0490 (4)	
H17	0.3162	0.4832	0.1979	0.059*	
N1	1.01637 (18)	0.41374 (11)	0.32770 (8)	0.0479 (3)	
N2	0.5647 (2)	0.21792 (11)	0.28725 (9)	0.0577 (4)	
O1	0.90826 (15)	0.43321 (10)	0.23971 (7)	0.0530 (3)	
O2	0.57595 (15)	0.38915 (8)	0.47380 (7)	0.0463 (3)	
Cl1	0.12337 (10)	0.36499 (4)	−0.09593 (3)	0.0808 (2)	

### Atomic displacement parameters (Å^2^)

**Table:**

	*U*^11^	*U*^22^	*U*^33^	*U*^12^	*U*^13^	*U*^23^
C1	0.0393 (7)	0.0430 (7)	0.0311 (6)	−0.0037 (5)	0.0038 (5)	−0.0036 (5)
C2	0.0611 (10)	0.0557 (9)	0.0320 (7)	−0.0063 (7)	0.0085 (6)	−0.0012 (6)
C3	0.0750 (12)	0.0552 (10)	0.0329 (7)	−0.0091 (8)	−0.0050 (7)	0.0026 (6)
C4	0.0534 (9)	0.0536 (9)	0.0478 (8)	−0.0034 (7)	−0.0161 (7)	0.0026 (7)
C5	0.0364 (7)	0.0490 (8)	0.0469 (8)	−0.0039 (6)	−0.0034 (6)	−0.0004 (6)
C6	0.0344 (6)	0.0382 (7)	0.0330 (6)	−0.0043 (5)	0.0005 (5)	−0.0019 (5)
C7	0.0275 (6)	0.0414 (7)	0.0353 (6)	−0.0025 (5)	0.0041 (5)	−0.0016 (5)
C8	0.0282 (6)	0.0424 (7)	0.0279 (5)	−0.0019 (5)	0.0040 (4)	−0.0002 (5)
C9	0.0333 (6)	0.0547 (8)	0.0323 (6)	0.0039 (6)	0.0059 (5)	−0.0008 (6)
C10	0.0384 (7)	0.0481 (8)	0.0273 (5)	−0.0052 (6)	0.0040 (5)	0.0014 (5)
C11	0.0353 (7)	0.0451 (7)	0.0343 (6)	−0.0034 (5)	0.0073 (5)	0.0030 (5)
C12	0.0418 (7)	0.0389 (7)	0.0298 (6)	−0.0008 (5)	0.0064 (5)	0.0056 (5)
C13	0.0528 (9)	0.0471 (8)	0.0391 (7)	0.0062 (6)	0.0120 (6)	0.0014 (6)
C14	0.0770 (12)	0.0459 (9)	0.0366 (7)	0.0024 (8)	0.0102 (7)	−0.0040 (6)
C15	0.0699 (11)	0.0439 (8)	0.0331 (7)	−0.0089 (7)	−0.0049 (7)	0.0075 (6)
C16	0.0475 (9)	0.0734 (11)	0.0413 (8)	0.0023 (8)	−0.0009 (6)	0.0090 (7)
C17	0.0445 (8)	0.0679 (10)	0.0334 (7)	0.0071 (7)	0.0049 (6)	0.0014 (6)
N1	0.0330 (6)	0.0693 (9)	0.0407 (6)	−0.0043 (6)	0.0055 (5)	0.0062 (6)
N2	0.0748 (10)	0.0515 (8)	0.0436 (7)	−0.0157 (7)	0.0038 (7)	−0.0009 (6)
O1	0.0353 (5)	0.0865 (9)	0.0387 (5)	−0.0052 (5)	0.0106 (4)	0.0121 (5)
O2	0.0362 (5)	0.0721 (7)	0.0316 (5)	0.0020 (5)	0.0095 (4)	0.0032 (4)
Cl1	0.1073 (5)	0.0743 (4)	0.0450 (3)	−0.0153 (3)	−0.0245 (3)	0.0034 (2)

### Geometric parameters (Å, º)

**Table:**

C1—O2	1.3750 (17)	C9—H9A	0.9700
C1—C2	1.3863 (19)	C9—H9B	0.9700
C1—C6	1.396 (2)	C10—N2	1.139 (2)
C2—C3	1.384 (3)	C11—O1	1.4477 (17)
C2—H2	0.9300	C11—C12	1.5001 (18)
C3—C4	1.373 (3)	C11—H11	0.9800
C3—H3	0.9300	C12—C13	1.378 (2)
C4—C5	1.380 (2)	C12—C17	1.388 (2)
C4—H4	0.9300	C13—C14	1.388 (2)
C5—C6	1.3953 (19)	C13—H13	0.9300
C5—H5	0.9300	C14—C15	1.373 (3)
C6—C7	1.4509 (18)	C14—H14	0.9300
C7—N1	1.2731 (18)	C15—C16	1.375 (3)
C7—C8	1.5160 (17)	C15—Cl1	1.7380 (16)
C8—C10	1.4716 (19)	C16—C17	1.381 (2)
C8—C9	1.5264 (18)	C16—H16	0.9300
C8—C11	1.5398 (18)	C17—H17	0.9300
C9—O2	1.4361 (17)	N1—O1	1.4202 (16)
			
O2—C1—C2	116.15 (13)	C8—C9—H9B	109.6
O2—C1—C6	123.10 (12)	H9A—C9—H9B	108.1
C2—C1—C6	120.71 (14)	N2—C10—C8	178.78 (16)
C3—C2—C1	119.08 (15)	O1—C11—C12	111.15 (11)
C3—C2—H2	120.5	O1—C11—C8	102.89 (10)
C1—C2—H2	120.5	C12—C11—C8	116.37 (11)
C4—C3—C2	120.85 (15)	O1—C11—H11	108.7
C4—C3—H3	119.6	C12—C11—H11	108.7
C2—C3—H3	119.6	C8—C11—H11	108.7
C3—C4—C5	120.35 (15)	C13—C12—C17	118.99 (13)
C3—C4—H4	119.8	C13—C12—C11	122.50 (13)
C5—C4—H4	119.8	C17—C12—C11	118.51 (12)
C4—C5—C6	119.99 (15)	C12—C13—C14	120.73 (15)
C4—C5—H5	120.0	C12—C13—H13	119.6
C6—C5—H5	120.0	C14—C13—H13	119.6
C1—C6—C5	119.01 (13)	C15—C14—C13	118.96 (15)
C1—C6—C7	117.50 (12)	C15—C14—H14	120.5
C5—C6—C7	123.45 (13)	C13—C14—H14	120.5
N1—C7—C6	128.10 (12)	C14—C15—C16	121.56 (15)
N1—C7—C8	113.80 (12)	C14—C15—Cl1	119.25 (13)
C6—C7—C8	118.09 (11)	C16—C15—Cl1	119.19 (15)
C10—C8—C7	108.77 (11)	C15—C16—C17	118.86 (16)
C10—C8—C9	111.69 (11)	C15—C16—H16	120.6
C7—C8—C9	108.02 (10)	C17—C16—H16	120.6
C10—C8—C11	112.54 (11)	C16—C17—C12	120.89 (15)
C7—C8—C11	98.32 (10)	C16—C17—H17	119.6
C9—C8—C11	116.36 (12)	C12—C17—H17	119.6
O2—C9—C8	110.13 (11)	C7—N1—O1	108.55 (11)
O2—C9—H9A	109.6	N1—O1—C11	107.83 (10)
C8—C9—H9A	109.6	C1—O2—C9	117.36 (11)
O2—C9—H9B	109.6		
			
O2—C1—C2—C3	177.14 (15)	C9—C8—C11—O1	141.41 (12)
C6—C1—C2—C3	−0.6 (2)	C10—C8—C11—C12	33.88 (17)
C1—C2—C3—C4	0.8 (3)	C7—C8—C11—C12	148.26 (12)
C2—C3—C4—C5	−0.2 (3)	C9—C8—C11—C12	−96.82 (15)
C3—C4—C5—C6	−0.5 (3)	O1—C11—C12—C13	6.67 (19)
O2—C1—C6—C5	−177.67 (13)	C8—C11—C12—C13	−110.64 (16)
C2—C1—C6—C5	−0.1 (2)	O1—C11—C12—C17	−173.80 (13)
O2—C1—C6—C7	0.2 (2)	C8—C11—C12—C17	68.89 (18)
C2—C1—C6—C7	177.75 (13)	C17—C12—C13—C14	0.4 (2)
C4—C5—C6—C1	0.6 (2)	C11—C12—C13—C14	179.89 (14)
C4—C5—C6—C7	−177.06 (14)	C12—C13—C14—C15	−1.0 (2)
C1—C6—C7—N1	166.36 (15)	C13—C14—C15—C16	0.6 (3)
C5—C6—C7—N1	−15.9 (2)	C13—C14—C15—Cl1	179.77 (12)
C1—C6—C7—C8	−11.99 (18)	C14—C15—C16—C17	0.5 (3)
C5—C6—C7—C8	165.74 (13)	Cl1—C15—C16—C17	−178.73 (13)
N1—C7—C8—C10	99.59 (14)	C15—C16—C17—C12	−1.1 (3)
C6—C7—C8—C10	−81.84 (14)	C13—C12—C17—C16	0.7 (2)
N1—C7—C8—C9	−139.02 (14)	C11—C12—C17—C16	−178.84 (15)
C6—C7—C8—C9	39.55 (16)	C6—C7—N1—O1	−177.66 (13)
N1—C7—C8—C11	−17.73 (16)	C8—C7—N1—O1	0.74 (18)
C6—C7—C8—C11	160.85 (12)	C7—N1—O1—C11	18.43 (17)
C10—C8—C9—O2	63.03 (14)	C12—C11—O1—N1	−153.97 (12)
C7—C8—C9—O2	−56.54 (15)	C8—C11—O1—N1	−28.73 (14)
C11—C8—C9—O2	−165.87 (11)	C2—C1—O2—C9	162.27 (13)
C10—C8—C11—O1	−87.89 (13)	C6—C1—O2—C9	−20.0 (2)
C7—C8—C11—O1	26.48 (13)	C8—C9—O2—C1	48.97 (17)

### Hydrogen-bond geometry (Å, º)

**Table:**

*D*—H···*A*	*D*—H	H···*A*	*D*···*A*	*D*—H···*A*
C13—H13···O1	0.93	2.42	2.7733 (19)	102
C5—H5···O2^i^	0.93	2.47	3.3422 (19)	156

Symmetry code: (i) *x*+1, *y*, *z*.
